# NFκB (RelA) mediates transactivation of hnRNPD in oral cancer cells

**DOI:** 10.1038/s41598-022-09963-7

**Published:** 2022-04-08

**Authors:** Vikas Kumar, Anurag Kumar, Manish Kumar, Moien Rasheed Lone, Deepika Mishra, Shyam Singh Chauhan

**Affiliations:** 1 Department of Biochemistry, All India Institute of Medical Sciences, New Delhi, India; 2 Department of Biochemistry, All India Institute of Medical Sciences, Bilaspur, India; 3 Division of Oral Pathology, Centre for Dental Education and Research, All India Institute of Medical Sciences, New Delhi, India

**Keywords:** Molecular biology, Transcription, Oral cancer

## Abstract

Heterogeneous Ribonucleoprotein D (hnRNPD) is an RNA binding protein involved in post-transcriptional regulation of multiple mediators of carcinogenesis. We previously demonstrated a strong association of hnRNPD over expression with poor outcome in Oral Squamous Cell Carcinoma (OSCC). However, hitherto the precise molecular mechanism of its overexpression in oral cancer was not clear. Therefore, in an attempt to elucidate the transcriptional regulation of hnRNPD expression, we cloned 1406 bp of 5ʹ flanking region of human hnRNPD gene along with 257 bp of its first exon upstream to promoterless luciferase reporter gene in pGL3-Basic. Transfection of the resulting construct in SCC-4 cells yielded 1271 fold higher luciferase activity over parent vector. By promoter deletion analysis, we identified a canonical TATA box containing 126 bp core promoter region that retained ~ 58% activity of the full length promoter. In silico analysis revealed the presence of four putative NFκB binding motifs in the promoter. Sequential deletion of these motifs from the full-length promoter reporter construct coupled with luciferase assays revealed an 82% decrease in promoter activity after deletion of the first (−1358/−1347) motif and 99% reduction after the deletion of second motif (−1052/−1041). In-vivo binding of NFκB (RelA) to these two motifs in SCC-4 cells was confirmed by ChIP assays. Site directed mutagenesis of even one of these two motifs completely abolished promoter activity, while mutagenesis of the remaining two motifs had marginal effect on the same. Consistent with these findings, treatment of SCC-4 cells with PDTC, a known inhibitor of NFκB dramatically reduced the levels hnRNPD mRNA and protein. Finally, the expression of hnRNPD and NFκB in clinical specimen from 37 oral cancer patients was assessed and subjected to Spearmen’s Correlation analysis which revealed a strong positive correlation between the two. Thus, results of the present study for the first time convincingly demonstrate NFκB (RelA) mediated transcriptional upregulation of hnRNPD expression in oral cancer.

## Introduction

Head and Neck Squamous Cell Carcinoma (HNSCC) represents a diverse group of moderately aggressive malignancy^1^. Among globally prevalent cancers, it ranks sixteenth in the list of top thirty cancers sites worldwide^2^. The development of Oral Squamous Cell Carcinoma (OSCC) which represents the major subtype of HNSCC is a complex multistep process that occurs due to alteration in the proto oncogene and tumor suppressor genes^3^. PI3-AKT, NFκB and Wnt signaling pathways are invariably altered in this malignancy^4,5^. NFκB is a key transcription factor that controls the transcription of various genes involved in cellular proliferation, survival, tumorigenesis as well as immune and inflammatory responses^6,7^. Increase in the nuclear expression of NFκB protein is associated with metastasis and poor survival of oral cancer patients^8^. Conversely, the silencing of NFκB (RelA) gene expression brings about favorable trends in oral cancer cell survival^9^.

Heterogeneous nuclear Ribonucleoprotein D (hnRNPD), also known as Adenylate Uridylate rich RNA binding Factor 1 (AUF1) is an ARE (AU-Rich Element) binding protein that regulates the stability of mRNAs encoded by genes involved in tumorigenesis, cell cycle, senescence, proliferation and apoptosis^10–12^. HnRNPD over-expression has been reported in cancers of thyroid, colon, rectum, esophagus, stomach, breast, oral cavity and liver^13–19^. Consistent with these reports, we previously observed over-expression of hnRNPD in OSCC and established an association of its increased nuclear localization with poor outcome of the disease^20^. The cytokine IL-6 activates JAK2/STAT3 signaling pathway thereby transactivating hnRNPD expression through transcription factor STAT3 in breast stromal fibroblasts. Interestingly, hnRNPD stabilize IL-6 mRNA which in turn increase NFκB expression. In line with these observations silencing of hnRNPD expression leads to inhibition of IL-6/STAT3/NFκB positive feedback loop in these cells^21^. In type 1 diabetes mellitus, over expression of hnRNPD leads to apoptosis of pancreatic beta cells by decreasing the expression of anti-apoptotic proteins BCL2 and MCL1. Conversely, SiRNA mediated silencing of hnRNPD expression restores anti-apoptotic proteins and attenuates of NFκB transcription factor that results in survival of pancreatic beta cells^22,23^. However, till date no systematic study has been carried out to elucidate the molecular mechanism of hnRNPD over-expression in OSCC. In the present study, we cloned full length human hnRNPD promoter and mapped its transcription start site. Then by promoter deletion analysis, site-directed mutagenesis and ChIP assays established the role of NFκB in transcriptional up regulation of hnRNPD in oral cancer cells. We further corroborated these findings by demonstrating a positive correlation between NFκB and hnRNPD expression in OSCC tissue specimen.

## Experimental procedures

### Tissue specimens

After obtaining approval from the Institute Ethics Committee for Post Graduate Research, All India Institute of Medical Sciences, New Delhi, India (NO.IECPG-162/19.04.2018) previously prepared paraffin embedded tissue specimens were used in the present study as described in our previous publication^20^. Written informed consent was obtained from all participants and/or their legal guardian(s) before sample collection. All experiments were performed in accordance with guidelines issued by institutional ethics committee. A total of 37 oral cancer and 10 normal oral mucosal tissue specimens were utilized in the present study. Clinico-pathological parameters of OSCC specimens are presented in Supplementary Table S1.

### Cell culture

Oral squamous cell carcinoma (OSCC) cell lines SCC-4 and SCC-25, were obtained from American Type Culture Collection (ATCC, VA, USA). MDA-1986 cells was a kind gift from MD Anderson Cancer Centre (Houston, TX, USA). All cells were characterized by STR profiling. OSCC cells were grown in monolayer cultures in Dulbecco’s modified eagle medium (DMEM) (Gibco, CA, USA) supplemented with 10% fetal bovine serum (FBS) (Gibco, CA, USA), 1 mM l-glutamine, 100 μg/ml streptomycin and 100 U/ml penicillin in a humidified CO_2_ incubator (5% carbon-dioxide, 95% air) at 37°C.

### Cell transfections

For all transfections, 10^6^ cells were plated in each well of a six-well plate. Next day, cells were washed twice with serum-free DMEM before transfecting with 1 μg of control or test plasmid DNA using Lipofectamine 3000 (Invitrogen, CA, USA), according to the manufacturer’s protocol. After 48 h, cells were washed three times with ice cold PBS, lysed and the luciferase activity in the cell lysates were measured using a dual-luciferase reporter assay system (Promega, Madison, WI, USA). The pRL-TK vector containing the Renilla luciferase gene under HSV-TK minimal promoter (Promega, Madison, WI, USA) were co-transfected with each construct and served as an internal control for normalization for the transfection efficiency as described previously^24^.

### Mapping of the transcription initiation site

The transcription initiation site of human hnRNPD gene was mapped using a RLM-RACE assay kit (Invitrogen Corporation, CA, USA) according to manufacturer’s protocol. Four μg of decapped and dephosphorylated total cellular RNA extracted from SCC-4 cells was ligated with the adapter RNA using T4 RNA ligase. The ligated RNA was then reverse transcribed by Avian Myeloblastosis Virus (AMV) reverse transcriptase using oligo dT primer and subjected to primary PCR using forward 5ʹGeneRacer primer and reverse hnRNPD Race R1 gene specific primer. Then a secondary PCR was performed with 5ʹGeneRacer nested primer and reverse hnRNPD Race R2 gene specific prime using 1.0 µL of 1: 20 diluted primary PCR products as template. The PCR amplified fragment was gel purified, cloned into pCR4-TOPO vector and subjected to double stranded DNA sequencing by Sanger’s dideoxy chain termination method.

### Amplification and cloning of 5ʹ upstream region of hnRNPD gene

**Figure 1:**
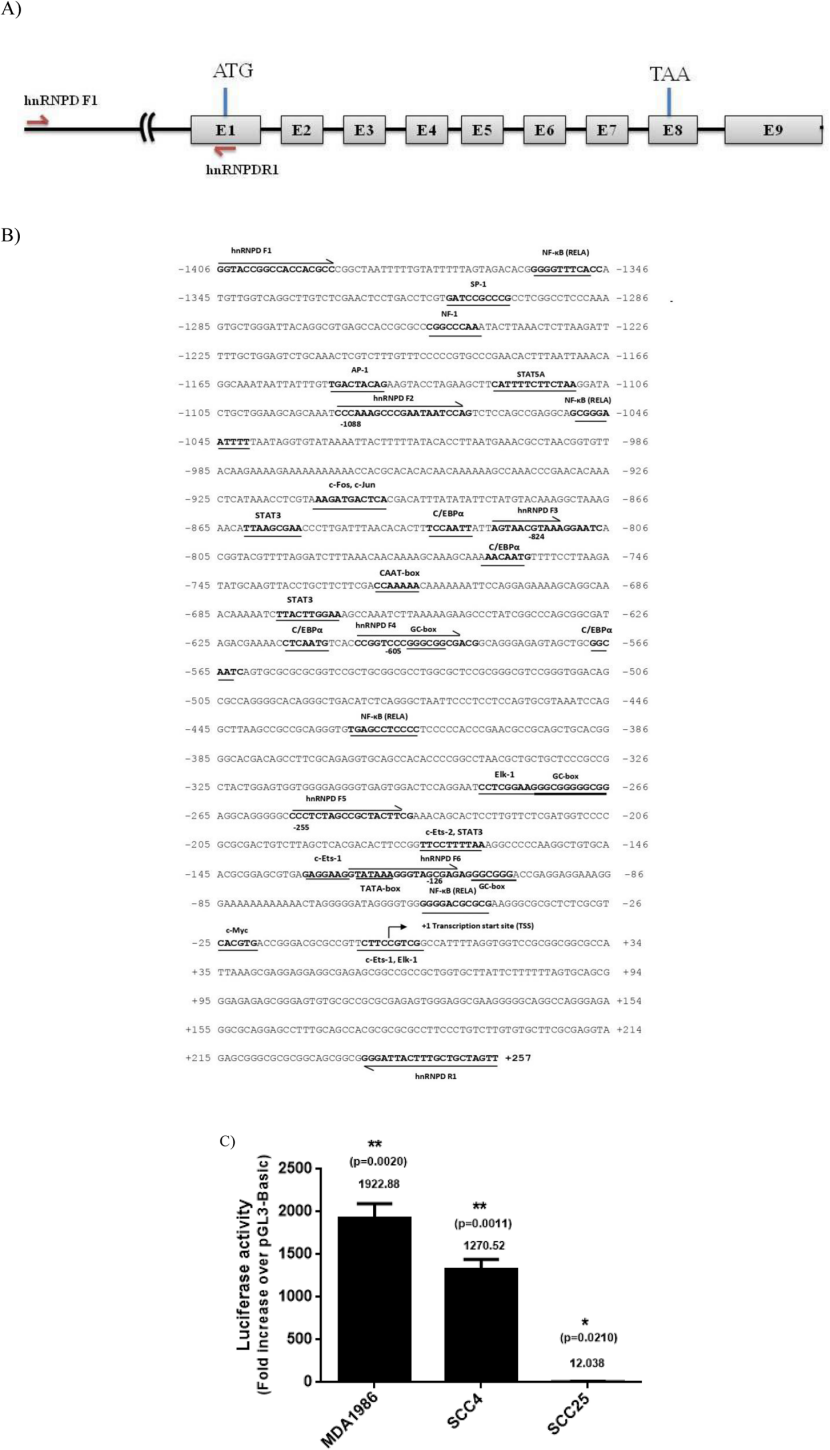
Cloning of human hnRNPD promoter and its nucleotide sequence analysis. (**A**) The human hnRNPD gene consists of 9 exons and 8 introns. Exons have been depicted as open boxes and numbered as E1 to E9 where as black bold lines between two boxes represents intron. The promoter region has been represented by a thin line and the forward and reverse primers used for its PCR amplification and cloning have been shown by arrows. The translation start codon (ATG) in E1 and translation stop codon (TAA) in the E8 have been marked by a vertical red line. The figure has not been drawn to scale (**B**) The 5ʹ-flanking region of the human hnRNPD gene along with 257 bp of first exon was PCR amplified and subjected to double stranded DNA sequencing to confirm its identity. The nucleotide sequence was analyzed using an online Transfec PROMO software (http://alggen.lsi.upc.es/cgi-bin/promo_v3/promo/promoinit.cgi?dirDB=TF_8.3). The putative potential transcription factors binding motifs have been shown in bold face and underlined. The primers used for generation of various promoter deletion constructs have been marked by arrows. The transcription start site mapped using RLM-RACE has been numbered as + 1 and marked with an arrow (↱). (**C**) The PCR amplified 5ʹ-flanking region of human hnRNPD gene was cloned upstream to the luciferase gene in pGL3-Basic and the resulting construct (pVKS-1), was co-transfected with pRL-TK plasmid in a three different oral cancer cell lines, MDA-1986 (Metastatic), SCC-4 and SCC-25 (Non-metastatic). After 48 h of transfection, the cells harvested and processed for dual luciferase assay. Renilla luciferase activity was used for normalization of transfection efficiency. Cells co transfected with pGL3-Basic and pRL-TK were processed identically and served as internal controls. Fold change in firefly luciferase activity in pVKS-1 transfected cells over the pGL3-Basic was plotted. The values are mean ± SD from three independent experiments performed in triplicate. The results were statistically analyzed using a paired two tailed Student’s t-test and values significantly different from each other have been marked by stars (**P ≤ 0.01, *P ≤ 0.05).

To PCR amplify the 5ʹ upstream region of hnRNPD gene, primers complementary to the hnRNPD gene sequence available in human genome data base (Accession no.NG_029103.1) were synthesized. Sites for NheI and MluI restriction endonucleases were incorporated in sense and antisense primers respectively to facilitate directional cloning of the amplified fragment. The nucleotide sequences of these primers have been given in supplementary Table 2 and their positions have been depicted in Fig. 1A. These primers were used to perform PCR using genomic DNA isolated from human Peripheral Blood Mononuclear cells as template. The PCR amplified fragment was gel purified and cloned into pGL3-Basic vector (Promega, Madison, WI, USA) upstream to the luciferase reporter gene. The resulting construct was subjected to double stranded DNA sequencing using pGL-forward (5ʹ-CTAGCAAAATAGGCTGTCCC-3ʹ) and pGL-reverse (5ʹ-CTTTATGTTTTTGGCGTCTTCC-3ʹ) universal sequencing primers. This construct was named as pVKS-1 and served as template for generation of promoter deletion constructs as well as for site directed mutagenesis (SDM) of specific transcription factor binding motif(s) (Supplementary Tables S2 and S3).

### Site directed mutagenesis

NFκB binding motifs in hnRNPD promoter were mutated using PCR based Quik Change II XL site-directed mutagenesis kit (Agilent Technologies, USA). Briefly, PCR was performed using the wild type promoter reporter constructs (pVKS-1) as template and synthetic oligonucleotides containing the desired mutation. The amplified PCR products were subjected to *DpnI* digestion to remove the parent template plasmid from the mixture. Remaining mixture containing the PCR amplified mutant plasmids was used to transform the competent E. *coli* cells and plated on Ampicillin containing LB Agar plates followed by incubation at 37°C. Next day individual colonies were picked, grown overnight in LB broth containing 50 µg/ml ampicillin and processed for plasmid isolation. The mutations were confirmed by restriction digestion followed by DNA sequencing.

### Western blotting

SCC-4 cells were washed twice with ice cold PBS and lysed with RIPA buffer. The cell lysate was centrifuged to remove the cell debris. An aliquot of clear supernatant containing 60 µg of protein was resolved onto 10% SDS-PAGE followed by transfer to 0.2um PVDF membrane (10,600,021, GE Healthcare, IL, USA). The blots were incubated with rabbit monoclonal anti-hnRNPD antibody (12382, Cell signaling technology, MA, USA), mouse monoclonal anti-RelA (17–10060, Sigma-Aldrich, MO, USA) or mouse monoclonal anti-β-Actin (SC47778, Santa Cruz Biotechnology, TX, USA), followed by incubation with HRP labeled IgG antibody (DAKO Cytomation, Glostrup, Denmark). Protein bands where visualized by ECL substrate (Pierce ECL Western Blotting Substrate, Thermo Fisher, MA, USA). In some experiments, SCC-4 cells subjected to sub cellular fractionation by using Nuclear and Cytoplasmic Extraction kit (#786-182, GBiosciences, St. Louis, USA), followed by western blotting.

### Chromatin immunoprecipitation assay (ChIP)

In vivo binding of NFκB to hnRNPD promoter was confirmed by ChIP assay^25^ using Imprint Chromatin Immunoprecipitation Kit (Sigma-Aldrich, St. Louis, MO, USA) according to the manufacturer’s protocol. Two µg of RelA antibody diluted in the 100 µl of antibody dilution buffer was incubated for 90 min in the strip wells provided in the kit. Simultaneously, 10^6^ SCC-4 PDTC treated and untreated cells were fixed with 1% formaldehyde for 10 min at 25°C to cross link the existing DNA–protein complex(s). Then the cells were treated with 125 mM glycine solution to quench crosslinking and processed for the isolation of nuclei. The nuclear pellet was resuspended in the shearing buffer provided in the kit and subjected to sonication using a Misonix sonicator at a power setting of 1.5 and a 100% duty cycle, for three 10 s pulses, with two minutes on ice in between pulses. Then the cell debris was removed by centrifugation at 14,000×*g* for 10 min at 4°C and clear supernatant containing sheared chromatin was transferred into the antibody pre-coated wells and incubated for 90 min. The immunoprecipitated DNA was recovered and used as template for PCR using ChIPF and ChIPR as sense and antisense primers complementary to the region flanking the NFκB motifs on human hnRNPD promoter (Supplementary Table S4). The PCR products were resolved on agarose gel, purified and sequenced. PCR performed with same primers using sheared chromatin DNA before immuno-precipitation served as input control. Similarly, chromatin immunoprecipitated using normal mouse IgG and anti-pol-II antibody were also used as template to perform PCR using the same primer set and served as controls.

### Real-Time PCR

Total RNA was extracted from a control and treated SCC-4 cells with Trizol reagent (Invitrogen, CA, USA) as described previously^20^. The quality and yield of the isolated RNA was assessed spectrophotometrically and the expression of hnRNPD was quantified by real-time PCR (RT-qPCR) using hnRNPD ORF F: GCCTTTCTCCAGATACACCTGAAG; hnRNPD ORF R: CT TATTGGTCTTGTTGTCCATGGG as forward and reverse primers respectively. Total RNA (1 μg) was reverse-transcribed using Reverse transcriptase (Thermo Scientific, Waltham, MA, USA) using random primers according to the manufacturer’s instructions. Real-time PCR reactions were performed and quantified by Maxima SYBR Green (Thermo Scientific, Waltham, MA, USA) using CFX96 Touch Real- Time PCR Detection System (BioRad, Hercules, CA, USA) using the ribosomal 18S RNA (18S ribosomal–Forward: GTAACCCGTTGAACCCCATT; Reverse: CCA TCCAATCGGTAGTAGCG) as an internal control for normalization. Details of primers used in this experiment are listed in Supplementary Table S5. All assays were performed in triplicate in a 10 µL two-step reaction. The specificity of the amplified PCR products was assessed by melting curve analysis and agarose gel electrophoresis of a small aliquot of the reaction followed by staining with ethidium bromide as described previously^20^.

### Confocal laser scanning microscopy (CLSM)

CLSM was performed as described previously^20^. Briefly, 5 × 10^4^ SCC-4 cells were plated on cover slips. After 24 h, the cells were washed with phosphate buffered saline (PBS, 0.01 M, pH = 7.2) and fixed in acetone: methanol mixture (1:1) at −20°C for 20 min. Cells were washed and permeabilized with 0.1% Tween in PBS, followed by blocking with 5% BSA for 1 h. Then they were incubated with mouse monoclonal anti-RelA antibody (17-10060, Sigma-Aldrich, MO, USA) overnight at 4 °C. Expression of RelA was detected by using fluorescein isothiocynate (FITC)-labeled goat anti-mouse secondary antibody (#62-6511, Invitrogen Corporation, CA, USA) and mounted with fluoroshield mounting medium with DAPI (ab104139, Abcam, CA). Images were captured by using confocal laser scanning microscope (CLSM)-LSM510 scanning module (Nixcon, Microscopy, Jena GmbH, Japan).

### Immunohistochemistry

Paraffin-embedded tissue sections were deparaffinized followed by antigen retrieval, and quenching of endogenous peroxidase activity with hydrogen peroxide (0.3% v/v). Then the non-specific binding was blocked with 1% bovine serum albumin (BSA). The tissue sections were then incubated with either rabbit monoclonal anti-hnRNPD antibody or mouse monoclonal anti-RelA antibody for 16 h at 4°C. The primary antibody was detected using the Dako Envision kit (Dako CYTOMATION, Glostrup, Denmark) with diaminobenzidine as the chromogen and counterstained with hematoxylin. The sections were evaluated by light microscopy and scored using a semi-quantitative scoring system for both staining intensity (nuclear/cytoplasmic) and percentage positivity. The tissue sections were scored based on the % of immunostained cells as: 0–10% = 0; > 10–30% = 1; > 30–50% = 2; > 50–70% = 3 and > 70–100% = 4. Sections were also scored semi-quantitatively on the basis of staining intensity as negative = 0; mild = 1; moderate = 2; intense = 3. Finally, a total score was obtained by adding the score of percentage positivity and intensity giving a score range from 0 to 7 as described previously^20^.

### Statistical analysis

Statistical comparison between two groups was performed using Student’s *t*-test. The Spearman’s correlation analysis was carried out using GraphPad Prism 6 software (Graphpad Software, San Diego CA, USA).

## Results

### Cloning of human hnRNPD promoter and its nucleotide sequence analysis

Human hnRNPD gene located on chromosome 4q21.22 consists of 9 exons and 8 introns. It harbours translation start and stop codons in exon 1 and 8 respectively (Fig. 1A). To study the transcriptional regulation of human hnRNPD gene, we performed PCR amplification of 5ʹupstream region of human hnRNPD gene by using gene specific primers hnRNPD F1 and hnRNPD R1. The location and nucleotide sequence of these primers have been shown in Fig. 1B and Supplementary Table S2 respectively. The 1663 bp PCR product amplified from genomic DNA was cloned upstream to the luciferase reporter gene in promoterless pGL3-Basic plasmid to assess its promoter activity. The resulting hnRNPD promoter reporter construct was named as pVKS-1 and subjected to double stranded DNA sequencing using universal sequencing primers flanking the cloned fragment. Alignment of its nucleotide sequence with human genome sequence database using online NCBI nucleotide blast tool (https://blast.ncbi.nlm.nih.gov/) revealed 100% homology of its first 1406 bp with the 5ʹ upstream region of human hnRNPD gene and as expected the nucleotide sequence of the remaining fragment was identical to the first 257 bps of exon-1. By analyzing the nucleotide sequence of the putative promoter region using an online Transfec PROMO software (http://alggen.lsi.upc.es/cgi-bin/promo_v3/promo/promoinit.cgi?dirDB=TF_8.3). We identified potential binding motifs for transcription factors such as NFκB (RelA), C/EBPα, STAT3, ETS-1 and Elk-1. Furthermore, this high GC content (55%) region also contained a TATA box (−125), a CAAT box (−722) and three GC boxes. To establish the identity of cloned 5ʹ upstream region as a functional promoter, we transfected pVKS-1 in three different cell lines derived from human oral cancer namely MDA-1986, SCC-4 and SCC-25 cells and assayed luciferase activity in the lysates after 48 h of transfection. Surprisingly, we observed significantly higher transfection efficiency in SCC-4 cells as compared to MDA-1986 and SCC-25 cells (Supplementary Fig. S1A). However, after normalization for the transfection efficiency as shown in Fig. 1C, the luciferase activity turned out to be significantly higher in MDA 1986 cells (1922 fold over pGL-3 basic) as compared to SCC-4 (1270 fold over pGL-3 basic) and SCC-25 cells (12 fold over pGL-3 basic). These results conclusively demonstrate that the 5ʹupstream region of hnRNPD gene contained in pVKS-1 upstream to the luciferase reporter gene is indeed a functional promoter. Considering the fact that SCC-4 displayed highest transfection efficiency, all subsequent studies on characterization of hnRNPD promoter were carried out in these cells.

### Mapping of human hnRNPD gene transcription start site

**Figure 2:**
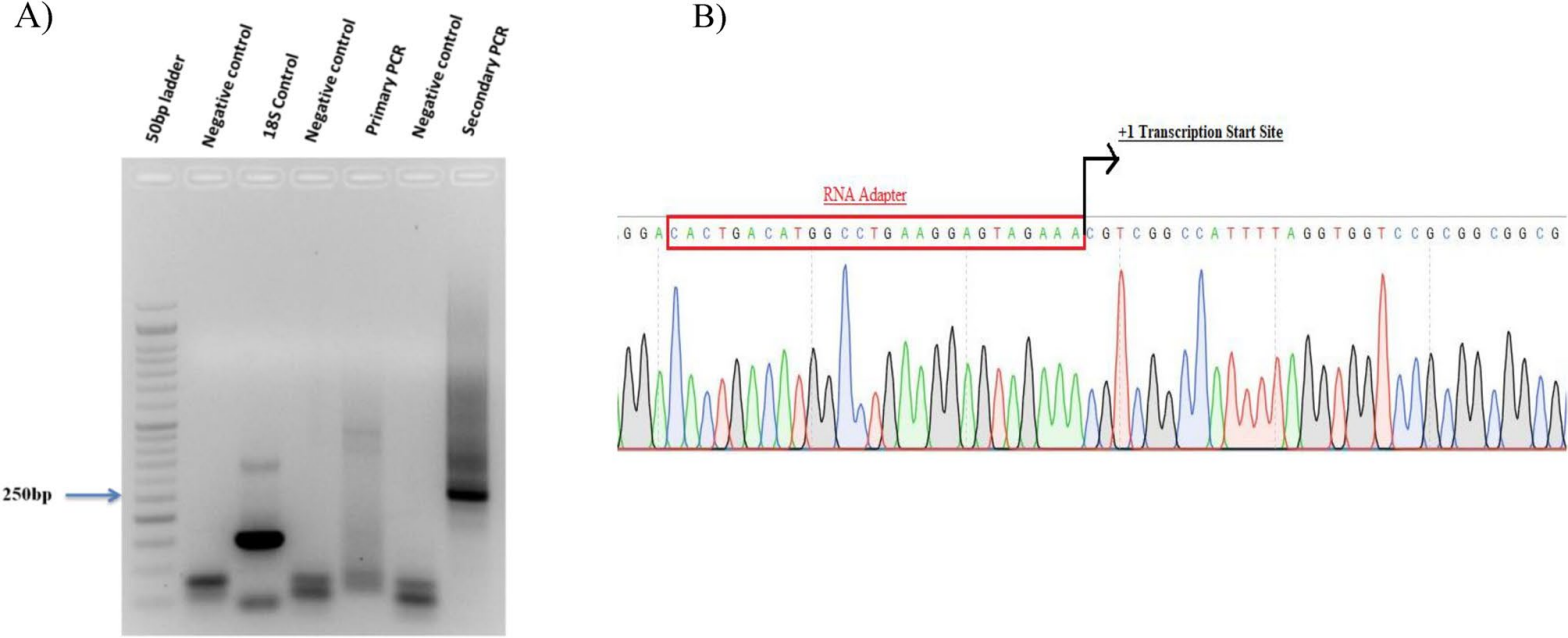
Mapping of of hnRNPD transcription start site in oral cancer cells. (**A**) RLM-RACE assay was used for mapping the transcription initiation site of human hnRNPD gene. Total RNA isolated from SCC-4 cells after treatment with Calf Intestinal Phosphatase (CIP) was decapped with tobacco acid pyrophosphatase. Then GeneRacer RNA adaptor was ligated to the 5ʹend of mRNA using T4 RNA Ligase. The adaptor ligated mRNA was reverse transcribed and subjected to primary and secondary PCR using nested adaptor and gene specific primers. 18S RNA was reverse transcribed and used as positive control and PCR reaction without template served as negative control. The PCR products were reolved on 1.2% agarose gel. (**B**) The prominent 250 bp band obtained after secondry PCR was subjected to double strand DNA sequencing after clonning into TA cloning vector pCR4. The first nucleotide after the RNA adaptor sequence was considered the transcription start site and has been marked as + 1.

To rule out the presence of promoter in the 257 bp of first exon in the amplified fragment and define the downstream end of the promoter we sought to map the transcriptional start site of human hnRNPD gene using SCC-4 cells by RNA Ligase Mediated RACE (RLM-RACE) assay. As shown in Fig. 2A, primary PCR using an RNA adaptor PCR primer and a gene specific primer lead to the amplification of two faint bands of 300 bp and 389 bp. However, secondary PCR using the nested adaptor and hnRNPD specific primers yielded a prominent 255 bp fragment and a faint 350 bp fragment (Fig. 2A). Amplification of no such fragments with any set of primers was observed when PCR was performed without any template (negative controls). The prominent fragment of 255 bp amplified after secondary PCR was cloned into the TA cloning vector pCR4. The six recombinant clones were randomly picked and processed for plasmid isolation followed by double stranded DNA sequencing using the universal primers flanking the cloning sites. As shown in Fig. 2B, 255 bp fragment in all six clones exhibited 100% homology to hnRNPD mRNA (accession no. NM_031369.2). In all these clones, cytosine was found to be the first nucleotide ligated to the adaptor primer thereby establishing the cytosine corresponding to the 314^th^ nucleotide upstream to the translation initiation site to be the major transcription initiation site. It has been shown in bold face and marked as + 1 in Fig. 1B.

### Deletion analysis of human hnRNPD gene promoter

**Figure 3:**
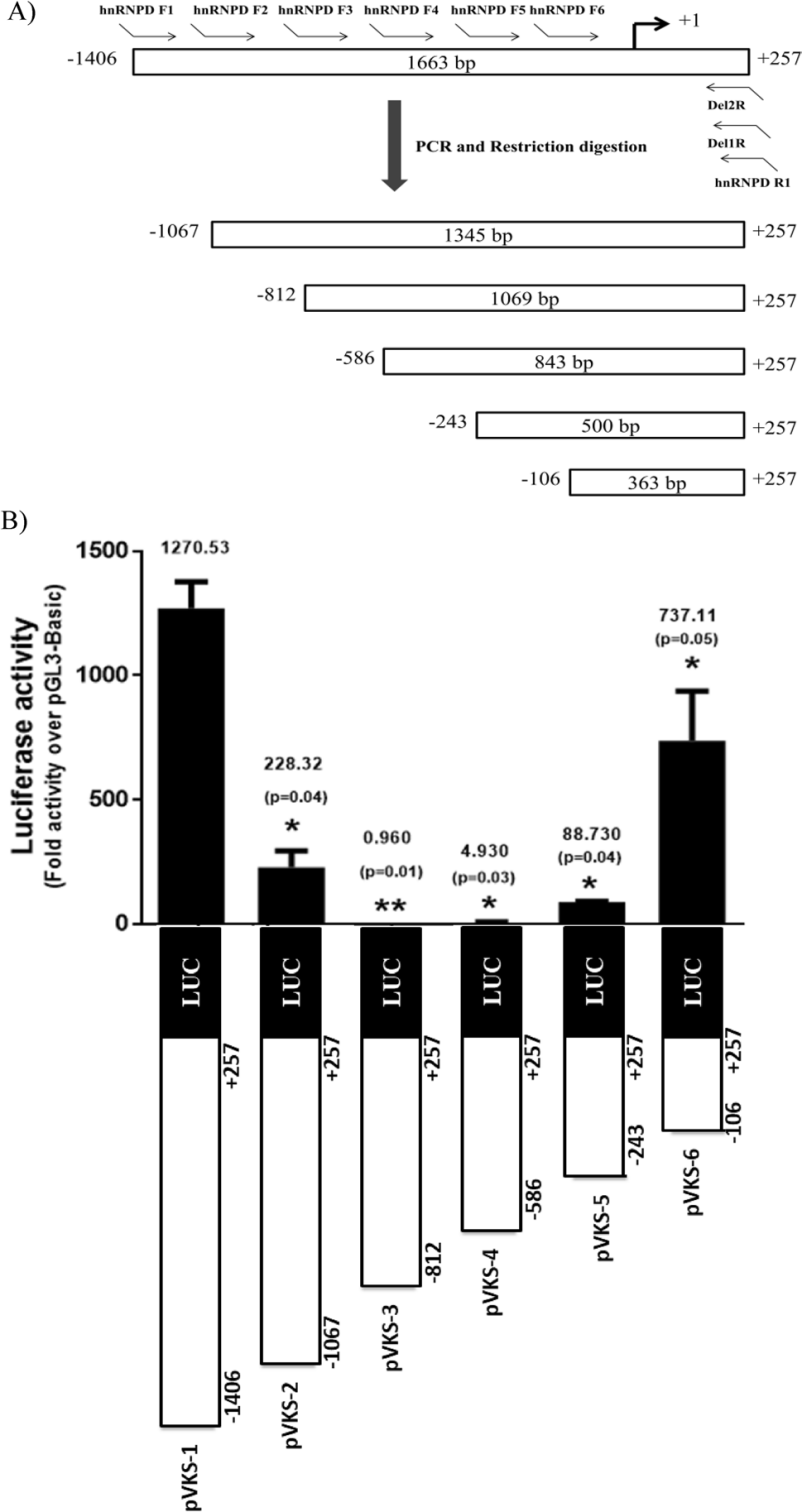
Deletion analysis of hnRNPD promoter. (**A**) Schematic diagram for generation of various deletions constructs of human hnRNPD promoter. (**B**) A series of promoter fragment having a common 3ʹ end (+ 257) but 5ʹ lacking increasing number of nucleotides (− 1088, − 824, − 605, − 255 and − 126) were PCR amplified as described in Materials and Methods and cloned separately upstream to luciferase reporter gene in pGL3-Basic plasmid to generate deletion constructs pVKS-2, pVKS-3, pVKS-4, pVKS-5 and pVKS-6 respectively. The individual constructs were co-transfected with pRL-TK plasmid in SCC-4 cells and the luciferase activities were measured 48 h post-transfection. Other details are same as described in Fig. 1. Values are mean ± SD from four independent experiments performed in triplicate. The results were statistically analyzed using a paired two tailed Student’s t-test and values significantly different from pVKS-1 have been marked by stars (**P ≤ 0.01, *P ≤ 0.05).

In order to define the minimal promoter region and identify the functional transcription factor binding motif, we generated a series of 5ʹ promoter deletion reporter constructs (Fig. 3A). The upstream end of each of these promoter deletion constructs has been marked by (⇀) in Fig. 1B. As evident from Fig. 3B deletion of 318 bp from the 5ʹend of full length promoter (pVKS-2; − 1088/ + 257) lead to a dramatic and statistically significant decrease (82%, p < 0.042) in the promoter activity as compared to the full length promoter reporter construct pVKS-1. This 318 bp promoter fragment which was lacking in pVKS-2 contains single putative transcriptional factor binding motifs for NFκB (RelA), Elk-1, AP-1 and SP-1. A further deletion of 264 bp (pVKS-3; − 824/ + 257) from the 5ʹend of pVKS-2 resulted in a 17.9% (p < 0.011) decrease in the promoter activity as compared to pVKS-2. This construct (pVKS-2) retained only ~ 0.960 fold promoter activity over pGL3-Basic and lost 99.92% of promoter activity compared to pVKS-1. These 264 nucleotides (− 824/ + 257) contains single motif for NFκB (RelA), STAT3, C/EBPα, and Ets-1 each. Interestingly, sequential deletion of the promoter region beyond -824 bp leads to a gradual increase in promoter activity. As evident from the data presented in Fig. 3B, constructs lacking 219 bp (− 605/ + 257: pVKS-4) and of promoter from 5ʹend displayed only ~ 4.93 fold activity over pGL3 Basic. However, this activity increased to 88.73 fold upon deletion of additional 350 bp (− 255/ + 257: pVKS-5) from 5ʹend. Further deletion of 129 bp (− 126/ + 257: pVKS-6), lead to 83% increase (~ 737.11 fold over pGL3-Basic; p < 0.015 ) in promoter activity as compared to pVKS-5. This construct displayed 58% activity as compared to the full-length promoter reporter construct pVKS-1. The region between − 126 and + 257 contains core promoter elements such as TATA box therefore; we conclude it to be core promoter.

### Critical role of NFκB (RelA) binding motifs in hnRNPD promoter activity

**Figure 4:**
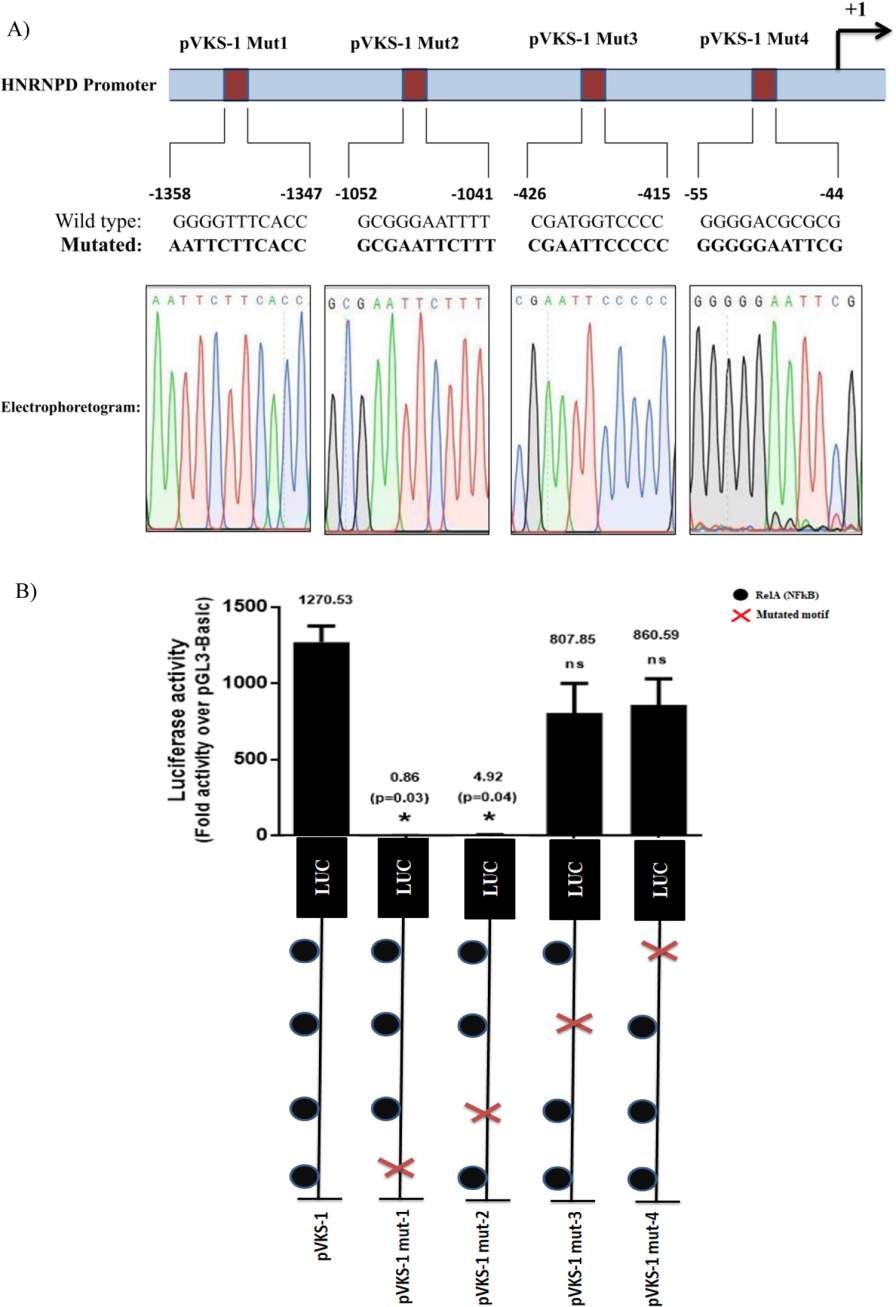
Functional relevance of NFκB binding motifs in human hnRNPD promoter. (**A**) Location and nucleotide sequence of the four putative NFκB (RelA) binding motifs as identified by in silico analysis of hnRNPD promoter. Each binding motif was individually subjected to site directed mutagenesis in promoter reporter construct pVKS-1 and the resulting constructs were named as pVKS-1 Mut1, pVKS-1 Mut2, pVKS-1 Mut3 and pVKS-1 Mut4 respectively. Mutation in each motif was confirmed by double stranded DNA sequencing and the mutated motifs have been shown in bold face along with their electrophoretogram. (**B**) Luciferase activity in SCC-4 cells after transfection with each mutant construct was assayed and compared with the activity of pVKS-1. Other details are given in Materials and Methods. Values are mean ± SD from four independent experiments performed in triplicate. Results were analyzed using a paired two tailed Student’s t-test and values significantly different from pVKS-1 marked by (*P ≤ 0.05).

By *in-silico* analysis we identified four putative NFκB (RelA) binding motifs. The location of these motifs in hnRNPD promoter have been marked in Fig. 1B and summarized in Fig. 4A. As shown in Fig. 3B and described in the preceding section, deletion of the promoter region containing the first motif (− 1358/− 1347; pVKS-2) lead to an 82% decrease in the promoter activity and it was completely abolished by deletion of the second motif (− 1052/− 1041; pVKS-3). These results suggest that the region between nucleotides − 1406 and − 824 is important for promoter activity. However, it was not clear whether the observed reduction in promoter activity was solely due to the deletion of NFκB binding motifs. Similarly, the effect of 3^rd^ NFκB binding motif (− 426/− 415) deletion could not be assessed by promoter deletion analysis as 99% of the activity was abolished by deletion of the promoter regions containing the first two motifs. Therefore, to systematically investigate the role of each NFκB binding motifs in hnRNPD expression, they were individually subjected to site-directed mutagenesis. The nucleotides changed in each of these motifs has been shown in Fig. 4A. The promoter reporter constructs harboring mutations in individual NFκB binding motifs were named as pVKS-1 Mut-1 (− 1358/− 1347), pVKS-1 Mut-2 (− 1052/− 1041), pVKS-1 Mut-3 (− 426/− 415) and pVKS-1 Mut-4 (− 55/− 44). All these constructs were transfected in SCC-4 cells to assess the effect of these mutations on promoter activity by measuring luciferase activity and the results have been presented in Fig. 4B. Consistent with the findings of promoter deletion analysis mutagenesis of first NFκB binding motif − 1358/− 1347 (pVKS-1 Mut-1) alone lead to ~ 99.93% (p < 0.0389) loss of promoter activity. Similarly, mutagenesis of the second motif located at − 1052/− 1041 (pVKS-1 Mut-2) leads to significant ~ 99.61% (p < 0.0391) decrease in promoter activity as compared to the wild type full length promoter reporter construct pVKS-1. However, no significant decrease in promoter activity was observed upon mutagenesis of third (− 426/− 415; pVKS-1 Mut-3) or fourth (− 55/− 44 (pVKS-1 Mut-4) NFκB binding motifs (Fig. 4B). From these results we conclude that first two NFκB binding motifs are essential for hnRNPD promoter activity.

### Inhibition of NFκB by Pyyrolidine dithiocarbamate (PDTC) dramatically decrease hnRNPD expression

**Figure 5:**
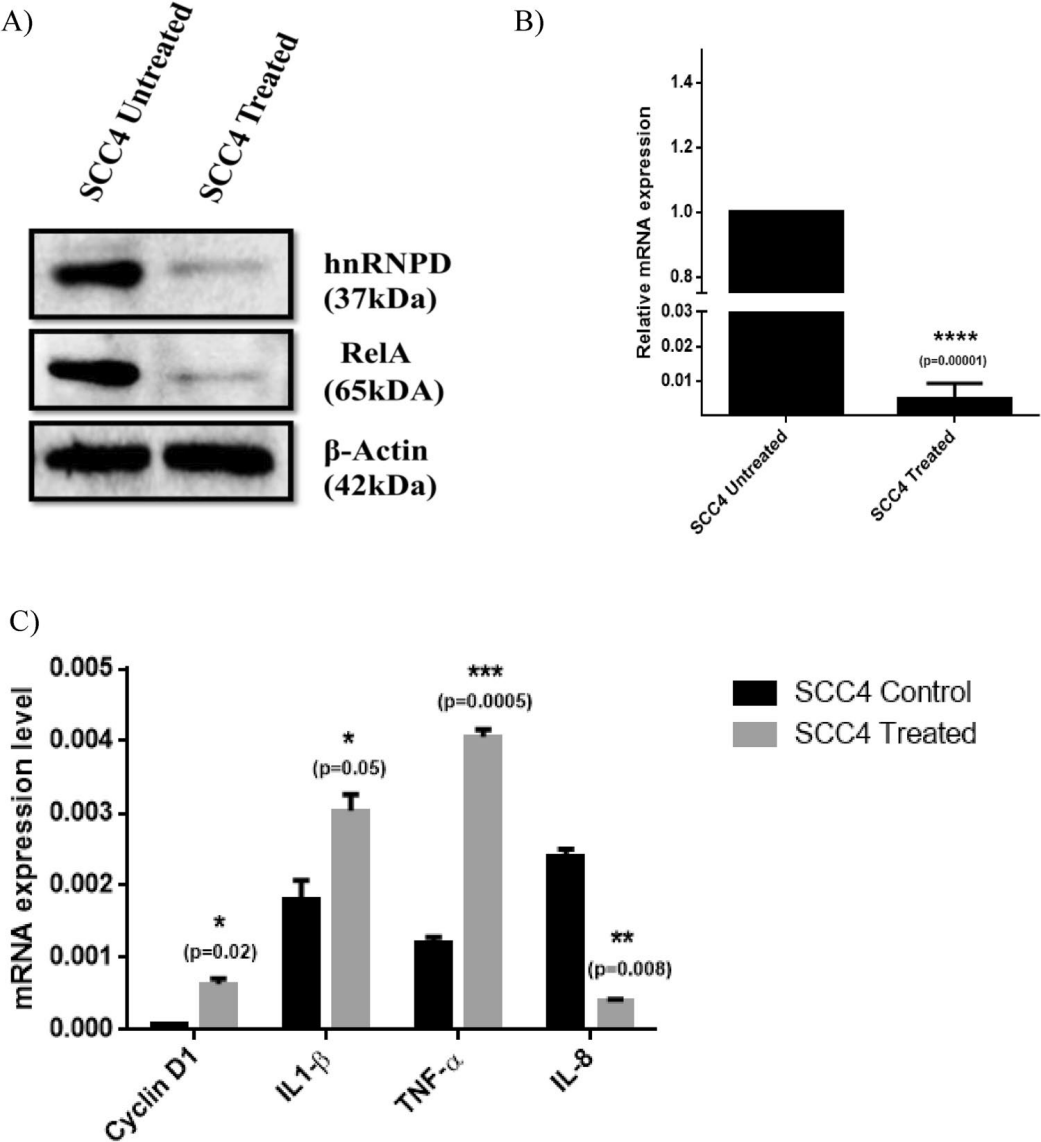
Reduction in hnRNPD mRNA and protein levels by pyrrolidine dithiocarbamates (PDTC) treatment in oral cancer cells. (**A**) Equal amount of total cell lysate proteins from PDTC treated and untreated SCC-4 cells were resolved on 10% SDS-PAGE and subjected Western blotting using monoclonal antibodies against hnRNPD or RelA. Western blot for β-actin served as internal control and was used for normalization for equal loading. (**B**) Total RNA isolated from SCC-4 cells before or after treatment with PDTC was reverse transcribed and subjected to real time PCR using specific hnRNPD primers. Simultaneously, the PCR was also performed using 18S ribosomal RNA specific primers and served as internal control for normalization of hnRNPD transcript. (**C**) Total RNA isolated from SCC-4 cells with or without treatment with PDTC was reverse transcribed and subjected to real time PCR using specific hnRNPD, Cyclin D1, TNF-α, IL1-β or IL-8 primers. 18S ribosomal RNA served as internal control for normalization. Values are mean ± SD from three independent experiments performed in triplicate. Results were analyzed using a paired two tailed Student’s t-test and values significantly different from untreated SCC-4 cells have marked by (*P ≤ 0.05, **P ≤ 0.01, ***P ≤ 0.001, ****P ≤ 0.0001).

Promoter deletion analysis and site directed mutagenesis suggest the key role of NFκB binding motifs in hnRNPD expression. To further corroborate these finding, we treated SCC-4 cells with PDTC, a well-known inhibitor of NFκB for 1 h followed by determining the expression of hnRNPD and NFκB. As shown in Fig. 5A, PDTC treatment dramatically reduced NFκB expression with concomitant and parallel reduction in the levels of immunoreactive hnRNPD in SCC-4 cells. In complete agreement with this observation we also observed a dramatic decrease (202 fold; p ≤ 0.0001) in hnRNPD mRNA levels (Fig. 5B). To further demonstrate the inhibition of hnRNPD by PDTC, we have assessed the levels of hnRNPD target mRNA such as Cyclin D1, IL-1β, and TNF-α (destabilized by hnRNPD)^10^ and IL-8 (stabilized by hnRNPD)^26^. As expected, inhibition of hnRNPD by PDTC led to a significant increase in the mRNA levels of Cyclin D1 (p < 0.02), IL-1β (p < 0.05), and TNF-α (p < 0.0005). On the contrary PDTC treated SCC-4 cells displayed significant reduction in mRNA levels of IL-8 (p < 0.008), as compared to the untreated cells. This further confirmed the inhibition of hnRNPD by PDTC (Fig. 5C). Thus our results unequivocally establish the key role of NFκB (RelA) in transcriptional regulation of hnRNPD expression.

### Binding of NFκB(RelA) to its cognate binding motifs on hnRNPD promoter

**Figure 6:**
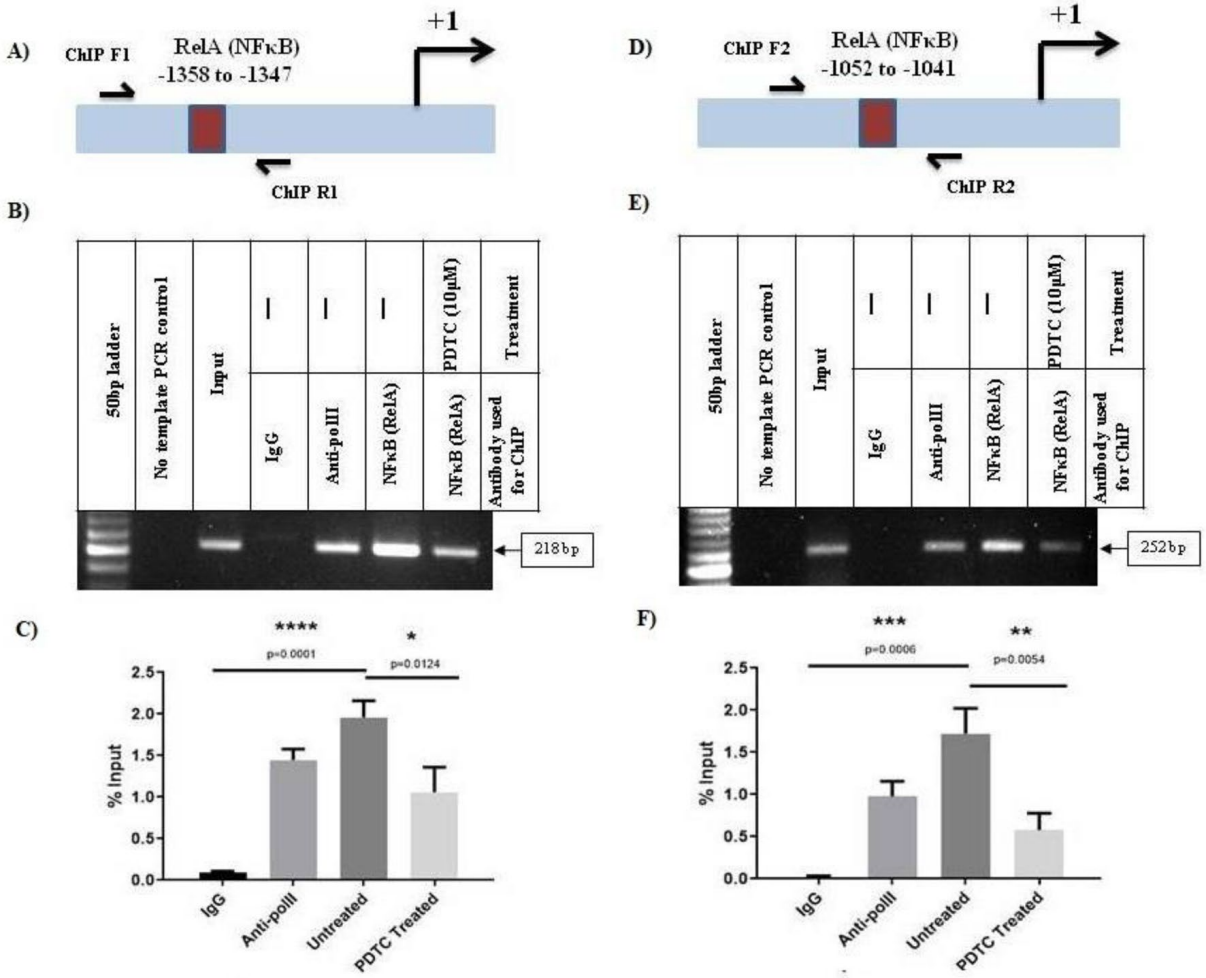
In vivo binding of NFκB (RelA) to its cognate motifs on human hnRNPD promoter. (**A**) Schematic representation depicting the positions of RelA (NFκB) binding motifs − 1358/− 1347 in the hnRNPD promoter and primers used for ChIP assay. Cross linked chromatin from PDTC treated or untreated SCC-4 cells was immunoprecipitated with anti-RelA antibody and subjected PCR after reversal of cross linking and sonication using the primers depicted in the supplementary Fig. S3. Sonicated genomic DNA from SCC-4 cells was used as input. Chromatin immunoprecipitated with Anti-pol II antibody was subjected to PCR using GAPDH primers and served as positive control. While PCR with gene specific primers and chromatin immunoprecipitated using normal mouse IgG served as negative control (IgG lane). Similarly, PCR performed with gene specific primers without a template also served as control. A 50 bp DNA ladder was used to determine the size of PCR fragments on agarose gel. (**B**) Representative agarose gel image of ChIP assay for RelA (NFκB) binding motifs − 1358/− 1347. (**C**) Densitometric quantification PCR fragment of ChIP assay for RelA (NFκB) binding motifs − 1358/− 1347. Values are mean ± SD from there independent experiments. Results were analyzed by Student’s t test and values significantly different from respective controls have been marked by stars (*P ≤ 0.05, ****P ≤ 0.0001). (**D**) Schematic representation depicting the position of RelA (NFκB) binding motif (− 1052/− 1041) in hnRNPD promoter) and primers used for ChIP assay. (**E**) Representative agarose gel image of ChIP assay for RelA (NFκB) binding motifs (− 1052/− 1041). (**F**) Densitometric quantification PCR fragment of ChIP assay for RelA (NFκB) binding motifs (− 1052/− 1041). Values are mean ± SD from three independent experiments. Values significantly different from respective controls have been marked by stars (**P ≤ 0.01, ***P ≤ 0.001). Other details are same as described for RelA (NFκB) binding motifs − 1358/− 1347.

We performed Chromatin Immunoprecipitation (ChIP) assays to study in-vivo binding of this transcription factor to its cognate motifs (− 1358/− 1347 and − 1052/− 1041) on hnRNPD promoter. The crosslinked chromatin from PDTC treated and an untreated SCC-4 cell was immunoprecipited with NFκB (RelA) antibody and subjected to PCR using gene specific primers flanking the abovementioned NFκB motifs (Fig. 6A,D). PCR was also performed with the same sets of primers using chromatin immunoprecipitated with normal mouse IgG and served as negative controls. Similarly, PCR performed using GAPDH gene specific primers and chromatin immunoprecipitated with Anti Pol II antibody served as positive control. As shown in Fig. 6B, use of chromatin immunoprecipitated with NFκB antibody from untreated SCC-4 and primers flanking − 1358/− 1347 NFκB motif lead to the amplification of an expected size fragment of 217 bp. Similarly, we observed the amplification of a 252 bp (expected size) fragment when the PCR was performed using the same chromatin template and the primers flanking the − 1052/− 1041 NFκB binding motif (Fig. 6E). The intensity of these bands was significantly reduced (p < 0.0124/Fig. 6C and p < 0.0054/Fig. 6F) when the same amount of NFκB antibody immunoprecipitated chromatin isolated from PDTC treated SCC-4 cells used as template (Fig. 6B,C,E,F). However, amplification of no such fragments could be seen with the same set of primers when IgG immunoprecipitated chromatin was as template (Fig. 6B,C,E,F). Similarly, no amplification of any size DNA fragment could be detected using hnRNPD ORF specific PCR primers and chromatin immunoprecipitated with NFκB antibody (Supplementary Fig. S2). These results convincingly demonstrate that NFκB specifically binds to its above-mentioned cognate motifs on hnRNPD promoter in oral cancer cells SCC-4 cells *in-vivo* and this binding is dramatically reduced/abrogated by PDTC treatment.

Finally, we performed sub cellular fractionation and Confocal laser scanning microscopy of SCC-4 cells to establish nuclear localization of NFκB (RelA) to further corroborate its role in transactivation of hnRNPD expression. Both these techniques confirm the presence of NFkB (RelA) in the nuclear compartment (Supplementary Fig. S1B,C).

### Positive correlation between hnRNPD and NFκB (RelA) expression in oral cancer

**Figure 7:**
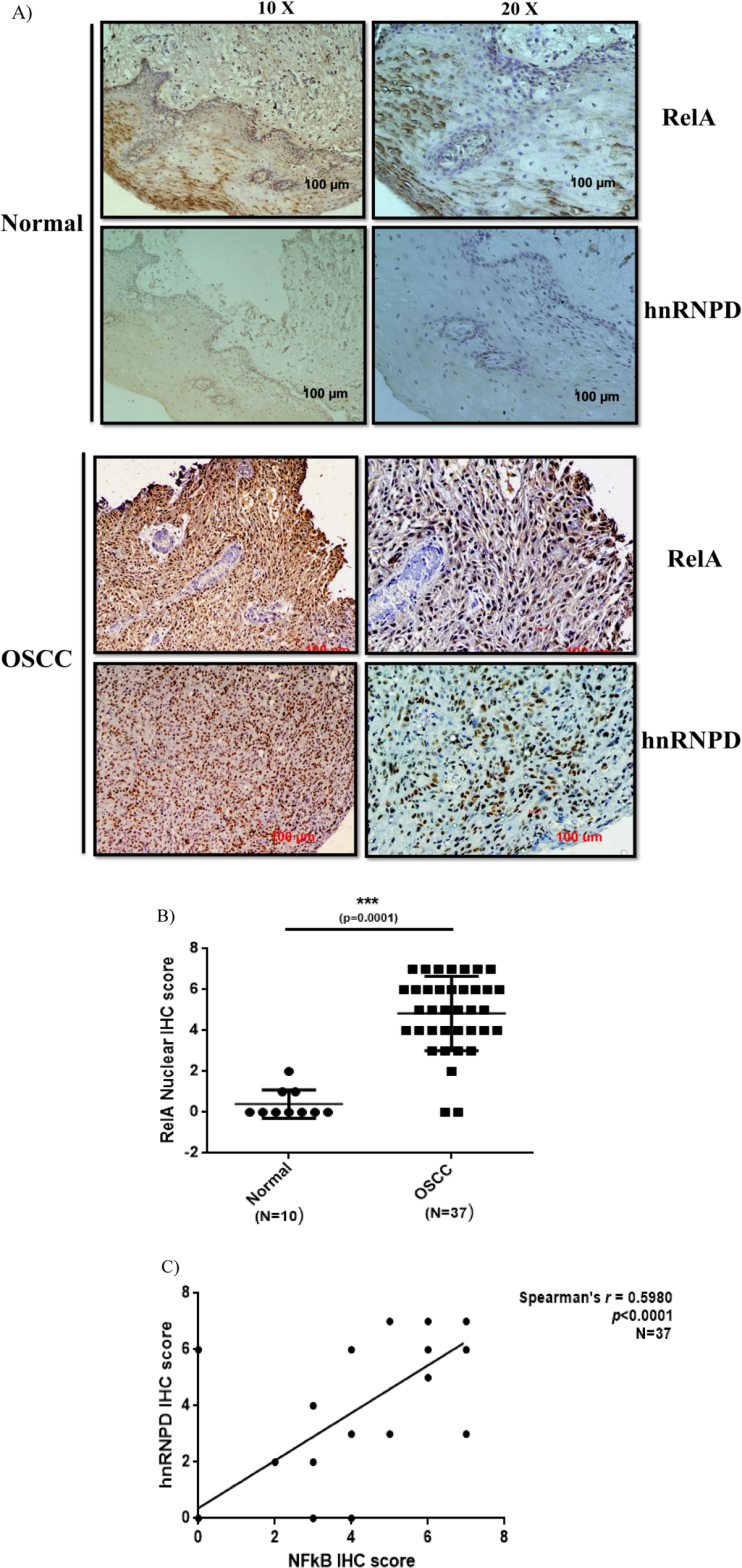
Expression of RelA and hnRNPD in oral cancer. Paraffin-embedded tissue sections of oral cancer were immunostained using anti-hnRNPD or ant-RelA antibody. The expression of both the proteins was scored independently by two pathologists blinded to the identity of sections and their score. (**A**) The representative images of normal and OSCC immunostained sections. (**B**) Nuclear expression of RelA in normal and OSCC tissue specimens. (**C**) Correlation between hnRNPD and NFκB expression in OSCC. Spearmen’s correlation analysis revealed a strong positive correlation between nuclear expression of hnRNPD and NFκB in OSCC.

In view of our findings on transcriptional up regulation of hnRNPD expression by NFκB in oral cancer cells, it was of interest to validate these results in clinical specimen of oral cancer patients. Therefore, we analyzed the expression of hnRNPD and NFκB (RelA) in clinical specimens from 37 oral cancer patients (N = 37) and oral mucosa from 10 normal subjects by immunohistochemistry. Clinical features of the patients used in the present study are given in Table S1. The representative photomicrograph of tissue sections immunostained for NFκB (RelA) and hnRNPD have been shown in Fig. 7A. In normal oral mucosa, mild cytoplasmic expression of NFκB was observed, whereas it was barely detectible in the nuclear compartment. However, the expression of hnRNPD was undetectable in cytosol and barely detectable in the nuclei in normal mucosa. On the other hand in oral cancer tissue specimens displayed elevated expression of hnRNPD and NFκB in nuclear compartment (Fig. 7A). Also as compared to the normal mucosa the nuclear expression of NFκB was found to be significantly higher (p < 0.0001) in OSCC specimens (Fig. 7B). Spearman’s correlation analysis revealed a strong positive (p < 0.0001) correlation (r = 0.5980) between hnRNPD and NFκB (RelA) expression in oral cancer tissue (Fig. 7C). These results further corroborate transactivation of hnRNPD expression by NFκB.

## Discussion

HnRNPD post transcriptionally regulate the expression of genes implicated in carcinogenesis. Its over-expression in various malignancies is extensively documented^13–20^. Consistent with these reports we previously observed elevated expression of hnRNPD in OSCC tissues samples and established its association with poor outcome of this disease^20^. However, very limited information was available regarding molecular mechanisms underlying its transcriptional upregulation. Therefore, in present study we sought to elucidate the molecular mechanism hnRNPD transcriptional up regulation in OSCC cells.

Previously, we successfully used PCR based strategy to clone and characterize human cathepsin L and dipeptidyl peptidase-III promoters^24,27^. The same strategy was employed in the present study to clone a 1663 bp 5ʹ upstream region of hnRNPD gene. The nucleotide sequence of this fragment exhibited 100% homology with the 4q21 locus of human genome and 257 bp of its downstream region displayed perfect match with the 5ʹ UTR of hnRNPD mRNA (accession no. NM_031369.2). This fragment displayed varying promoter activity in three different oral cancer cells namely SCC-4, SCC-25 and MDA-1986. However, MDA-1986 cells displayed highest promoter activity. This was in agreement with our previous report on the levels hnRNPD protein in these cells^20^. Elevated hnRNPD promoter activity has also been reported in breast cancer cells^17^. By nucleotide sequence analysis, we identified a TATA box, CAAT box and multiple GC boxes in hnRNPD promoter. TATA box is characteristic feature of highly regulated genes^28^. Approximately 32% of human genes contain TATA box, they are differentially expressed and induced by stress^29,30^. The expression of hnRNPD is differentially expressed at various stages of embryonic development in mice as well as its expression is induced by stress^31–33^. Thus the presence of TATA box in hnRNPD promoter conforms to its differential expression and stress inducibility. By 5ʹRACE assays we mapped the transcription initiation site 313 bp upstream to translation initiation codon. As no other report on mapping of transcription initiation site is available in the literature present study for the first time identified this site in the hnRNPD gene.

Deletion of hnRNPD promoter region (− 1406 /− 1067) which contains putative binding motifs for transcription factors such as FOXP3, Elk-1, Sp-1 and NFκB (RelA) lead to a dramatic decrease (82%) in promoter activity (Fig. 3B). Even the site mutagenesis of NFκB (RelA) binding motif present in this region resulted in similar decrease in promoter activity. These results conclusively established that the loss in promoter activity following deletion of promoter region (− 1406/− 1067) was solely due to the deletion of NFκB (RelA) binding motif. In addition to this NFκB (RelA) binding motifs hnRNPD promoter contains three other motifs (− 1052/− 1041 and − 426/415; − 55/− 44) for this transcription factor. Of these three motifs mutagenesis of only (− 1052/− 1041) motif abolished 99% of the promoter activity while the mutations of other two motifs (− 426/415; − 55/− 44) had no effect on promoter activity. Thus we conclude that out the four, only two NFκB (RelA) binding motifs (− 1358/− 1347; − 1052/− 1041) are functionally active and both of them are essential for promoter activity. The functionality of both these motifs was further established by ChIP assays.

PDTC, a well-known NFκB inhibitor, is a thiol group containing antioxidant. It inhibits NFκB by two separate mechanisms; first it acts as a scavenger for free radicals and secondly it impedes the inhibitory subunit of IκB kinase thereby sequestering it within cytoplasm^34–36^. Treatment of oral cancer cells by PDTC resulted in drastic reduction in transcript and protein level of hnRNPD. These findings are in agreement with results of promoter deletion analysis, site directed mutagenesis and ChIP assay and thus corroborate the involvement of NFκB (RelA) in transcriptional upregulation of hnRNPD expression in OSCC. In this context it is noteworthy that over-expression and nuclear localization of NFκB (RelA) is associated with lymph node metastasis in OSCC^8^. In line with these reports confocal scanning microscopy and subcellar fractionation experiments revealed nuclear localization of NF-kB (RelA) in SCC-4 cells. Furthermore, we observed a strong positive correlation between nuclear localization of NFκB (RelA) and expression of hnRNPD in OSCC tissue samples.

**Figure 8:**
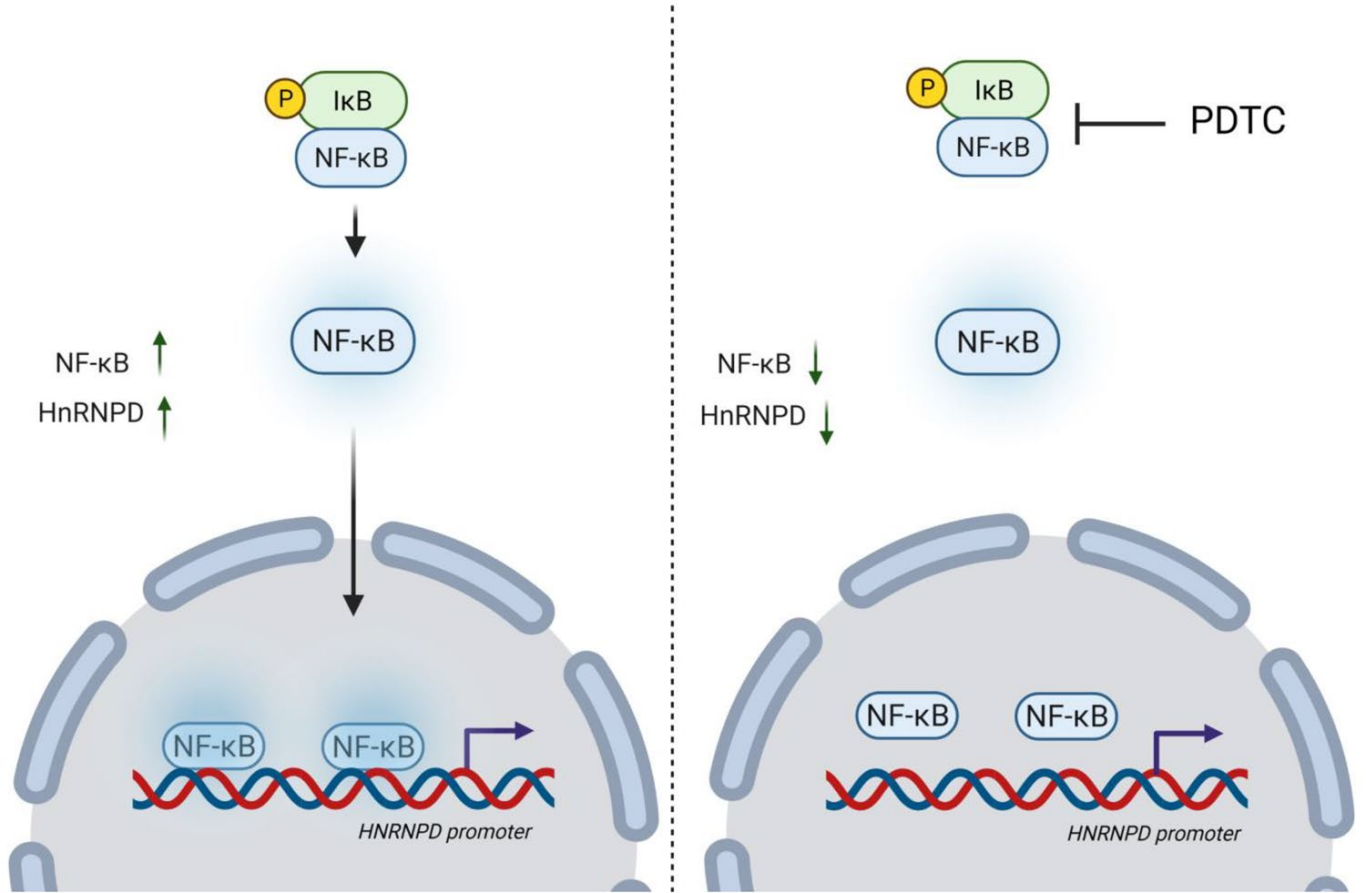
A schematic diagram of NFκB (RelA)-hnRNPD axis in oral cancer. Transcription factor NFκB (Rel A) binds to its binding motifs in hnRNPD promoter region and mediates its transcriptional upregulation in oral cancer cells. Inhibition of NFκB by PDTC leads to decrease in hnRNPD protein and transcript levels by inhibition of NFκB (RelA)-hnRNPD axis in oral cancer (image created with Biorender.com).

NFκB mediates the transcriptional up-regulation of genes involved in inflammation (IL-6 and TNF-α;^37,38^), cell adhesion (ICAM-1 and Tenascin-C;^39–41^), growth (IGFBP-1/2;^42,43^), and survival (Bcl-xL and BAX;^44,45^), which are the hallmarks of cancer^46^. Here we conclusivly demonstrate the transcriptional upregulation of hnRNPD by NFκB (RelA). The mechanism for the same has been summarized in Fig. 8. Anti-inflammatory agents which lower NF-kB levels have been used to control onset and progression of carcinogenesis^47–50^. Results of the present study taken together with our previous finding suggest that anti-inflammatory agents could be used to down regulate hnRNPD over-expression and hence improve the disease out come in OSCC.

## Conclusion

Present study, for the first time demonstrate the involvement of transcription factor NFκB (RelA) in transcriptional up regulation of human hnRNPD in oral cancer and suggest the role NFκB (RelA)-hnRNPD axis in oral cancer.

## Supplementary Information

Supplementary Information.

## Abbreviations

PI3K: Phosphatidylinositol-3 kinase
AKT: Protein kinase B
ARE: Adenylate uridylate rich element
IL-6: Interleukin-6
STAT3: Signal transducer and activator of transcription 3
JAK2: Janus kinase 2
BCL2: B cell lymphoma 2
MCL1: Myeloid cell leukemia 1
