# Supplementary material for: NFκB (RelA) mediates transactivation of hnRNPD in oral cancer cells
Source: Sci Rep. 2022 Apr 8;12:5944. doi: 10.1038/s41598-022-09963-7 (PMC8993925; doi:10.1038/s41598-022-09963-7)
Supplement: Supplementary file 1 — Supplementary Information. [file 41598_2022_9963_MOESM1_ESM.docx]

| **Patients No.** | **Age/Gender** | **Tumor site** | **Pathological**  **stage** | **Histological grade** |
| --- | --- | --- | --- | --- |
| \| 1 \| \| --- \| \| 2 \| \| 3 \| \| 4 \| \| 5 \| \| 6 \| \| 7 \| \| 8 \| \| 9 \| \| 10 \| \| 11 \| \| 12 \| \| 13 \| \| 14 \| \| 15 \| \| 16 \| \| 17 \| \| 18 \| \| 19 \| \| 20 \| \| 21 \| \| 22 \| \| 23 \| \| 24 \| \| 25 \| \| 26 \| \| 27 \| \| 28 \| \| 29 \| \| 30 \| \| 31 \| \| 32 \| \| 33 \| \| 34 \| \| 35 \| \| 36 \| \| 37 \| \|  \| \|  \| \|  \| \|  \| \|  \| | \| 75/M \| \| --- \| \| 55/M \| \| 40/F \| \| 52/F \| \| 45/M \| \| 15/M \| \| 75/M \| \| 49/M \| \| 52/F \| \| 60/M \| \| 33/M \| \| 35/M \| \| 71/M \| \| 55/M \| \| 29/M \| \| 38/M \| \| 35/M \| \| 57/M \| \| 38/M \| \| 35/M \| \| 55/F \| \| 45/M \| \| 39/M \| \| 55M \| \| 60/F \| \| 35/M \| \| 35/M \| \| 55/M \| \| 48/M \| \| 31/M \| \| 52/M \| \| 36/M \| \| 33/M \| \| 44/F \| \| 32/M \| \| 35/M \| \| 31/M \| \|  \| \|  \| \|  \| \|  \| | \| Tongue \| \| --- \| \| Buccal mucosa \| \| Hard palate \| \| Tongue \| \| Tongue \| \| Buccal mucosa \| \| Buccal mucosa \| \| Tongue \| \| Buccal mucosa \| \| Retromolar trigone \| \| Buccal mucosa \| \| Retromolar trigone \| \| Tongue \| \| Tongue \| \| Buccal mucosa \| \| Buccal mucosa \| \| Retromolar trigone \| \| Buccal mucosa \| \| Buccal mucosa \| \| Buccal mucosa \| \| Alveolar \| \| Alveolar \| \| Buccal mucosa \| \| Soft palate \| \| Buccal mucosa \| \| Retromolar trigone \| \| Retromolar trigone \| \| Alveolar \| \| Gingivobuccal sulci \| \| Tongue \| \| Tongue \| \| Buccal mucosa \| \| Tongue \| \| Tongue \| \| Alveolar \| \| Tongue \| \| Tongue \| \|  \| \|  \| \|  \| \|  \| | \| T3N0M0 \| \| --- \| \| T4N1M0 \| \| T2N2M0 \| \| T2N1M0 \| \| T2N2M0 \| \| T4N0M0 \| \| T4N1M0 \| \| T2N1M0 \| \| T4N1M0 \| \| T3N1M0 \| \| T4N1M0 \| \| T4N1M0 \| \| T2N2M0 \| \| T1N1M0 \| \| T2N1M0 \| \| T4N2M0 \| \| T4N0M0 \| \| T4N1M0 \| \| T2N0M0 \| \| T2N1M0 \| \| T4N1M0 \| \| T4N1M0 \| \| T4N1M0 \| \| T2N2M0 \| \| T3N1M0 \| \| T4N1M0 \| \| T4N0M0 \| \| T4N2M0 \| \| T2N0M0 \| \| T3N2M0 \| \| T2N0M0 \| \| T3N2M0 \| \| T4N2M0 \| \| T2N1M0 \| \| T4N2M0 \| \| T1N0M0 \| \| T4N0M0 \| \|  \| \|  \| \|  \| \|  \| | \| PDSCC \| \| --- \| \| MDSCC \| \| WDSCC \| \| MDSCC \| \| MDSCC \| \| WDSCC \| \| WDSCC \| \| WDSCC \| \| WDSCC \| \| WDSCC \| \| MDSCC \| \| MDSCC \| \| WDSCC \| \| MDSCC \| \| MDSCC \| \| PDSCC \| \| MDSCC \| \| MDSCC \| \| MDSCC \| \| MDSCC \| \| MDSCC \| \| WDSCC \| \| WDSCC \| \| WDSCC \| \| WDSCC \| \| MDSCC \| \| MDSCC \| \| MDSCC \| \| WDSCC \| \| WDSCC \| \| MDSCC \| \| MDSCC \| \| WDSCC \| \| WDSCC \| \| MDSCC \| \| PDSCC \| \| WDSCC \| |

**Table S1:** Clinicopathological characteristics of tissue specimens used in the study

| **Name** | **Sequence** | **Location in**  **the promoter** | **Deleted region**  **in the promoter** |
| --- | --- | --- | --- |
| hnRNPDF1  (Sense)  hnRNPDF2  (Sense)  hnRNPDF3  (Sense)  hnRNPDF4  (Sense)  hnRNPDF5  (Sense)  hnRNPDF6  (Sense)  hnRNPDR1  (Antisense)  DelR1  (Antisense)  DelR2  (Antisense) | 5’ TCAGT**ACGCGT**GGTACCGGC  CACCACGCC 3’  5' GCAGC**GGTACC**CAAAGCCC  GAATAATCCAG 3'  5’ CA**GGTACC**AGTAACGTAAAG  GAATCACG 3’  5’ CC**GGTACC**GGGCGGCGACG 3’  5' CA**ACGCGT**CCCTCTAGCCGC  TACTTCG 3'  5’ AGAG**ACGCGT**ATAAAGGGTA  GCGAGAGG 3’  5’ ATAGC**GCTAGC**AACTAGCA  GCAAAGTAATCCC3’  5’ CGG**CCATGG**CCGCGAATTCA  CTAGTGATC 3’  5' AGATCTCGAGCCCGGGCTAG 3' | -1406 to -1386  -1088 to -1066  -812 to -792  -586 to -567  -243 to -224  -106 to -86  -237 to -257 | -1406 to -1087  -1087 to -811  -811 to -585  -585 to -242  -242 to -105 |

**Table S2:** PCR primers used to for amplification of 5’ nucleotide fragment and to generate various promoter reporter deletion constructs

| **Name of**  **construct** | **Sense**  **primer** | **Antisense**  **primer** | **Length of cloned**  **fragment** | **Number**  **of deleted**  **bases** |
| --- | --- | --- | --- | --- |
| pVKS-1  pVKS-2  pVKS-3  pVKS-4  pVKS-5  pVKS-6 | hnRNPD F1  hnRNPDF2  hnRNPDF3  hnRNPDF4    hnRNPDF5  hnRNPDF6 | hnRNPD R1  Del2R  Del2R  Del2R  Del1R  Del1R | -1406/+257(1663bp)  -1088/+257(1345bp)  -812/+257(1069bp)  -586/+257(843bp)  -243/+257(500bp)  -106/+257(363bp) | 0  319  277  227  344  138 |

**Table S3:** Details of various constructs used in the deletion analysis of hnRNPD promoter

| **Name**  **of**  **construct** | **Primer** | **Restriction**  **site**  **inserted** |
| --- | --- | --- |
| **pVKS-1 mut1** | 5’ –TTTAGTAGACAC**GAATTC**TTCACCATGTTGGTCAGGC-3’  5’ –GCCTGACCAACATGGTGAA**GAATTC**GTGTCTACTAAA-3’ | **EcoRI** |
| **pVKS-1 mut2** | 5’-TAATTTTATACACCTATTAAAA**GAATTC**GCTGCCTCGGCTGGAGAC-3’  5’-GTCTCCAGCCGAGGCAGC**GAATTC**TTTTAATAGGTGTATAAAATTA-3’ | **EcoRI** |
| **pVKS-1 mut3** | 5’-TGAGCTAAGACAGTCGCGCGGGG**GAATTC**GAGAACAAGGAG-3’  5’-CTCCTTGTTCTC**GAATTC**CCCCGCGCGACTGTCTTAGCTCA-3’ | **EcoRI** |
| **pVKS-1 mut4** | 5' GGTGGGGGG**GAATTC**GAAGGGCGCGCTCTCGCGTC 3'  5' CGCCCTTC**GAATTC**CCCCCCACCCCTATCCCCCT 3' | **EcoRI** |
| **ChIPF1**  **(Sense)** | 5’-TGAGATTACAGGTACCGGCCACC-3’ | **Amplicon size**    218 bp |
| **ChIPR1**  **(Antisense)** | 5’-CAAAGACGAGTTTGCAGACTCCA-3’ |  |
| **ChIPF2**  **(Sense)** | 5’ GCCCGGCCCAAATACTTAAA 3’ | **Amplicon size**    252 bp |
| **ChIPR2**  **(Antisense)** | 5’ TTGTTGTGTGTGCGTGGTT 3’ |  |

**Table S4:** Details of the primer used to generate NFκB (RelA) mutant constructs and ChIP assay.

| **Name**  **of**  **primer** | **Primer sequence** | **Amplicon size**  **(bp)** | **Annealing Tm (°C)** |
| --- | --- | --- | --- |
| **hnRNPD ORF F**  **(Sense)** | 5’-GCCTTTCTCCAGATACACCTGAAG-3’ | 101 | 58 |
| **hnRNPD ORF R**  **(Antisense)** | 5’-CTTATTGGTCTTGTTGTCCATGGG-3’ |  |  |
| **IL8 F**  **(Sense)** | 5’-TGGTCCACTCTCAATCACTCTC -3’ | 127 | 60 |
| **IL8**  **(Antisense)** | 5’-AGCTCTGTGGAAGGTGCAGT -3’ |  |  |
| **TNFα**  **(Sense)** | 5’-GTTGTAGCAAACCCTCAAGCTG- 3’ | 151 | 60 |
| **TNFα**  **(Antisense)** | 5’-CCTTGAAGAGGACCTGGGAG-3’ |  |  |
| **IL1β**  **(Sense)** | 5’-CTGAGCTCGCCAGTGAAA -3’ | 202 | 60 |
| **ILβ**  **(Antisense)** | 5’-TCCATGGCCACAACAACT -3’ |  |  |
| **Cyclin D1**  **(Sense)** | 5’-GCTGCGAAGTGGAAACCATC-3’ | 135 | 60 |
| **Cyclin D1**  **(Antisense)** | 5’-CCTCCTTCTGCACACATTTGAA-3’ |  |  |

**Table S5:** Details of primers used for Real-Time PCR.

1.
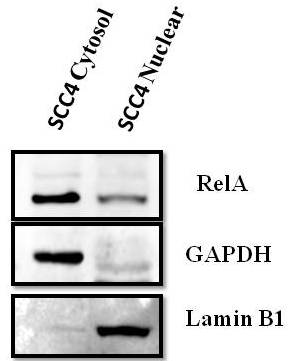
 **B)**

**
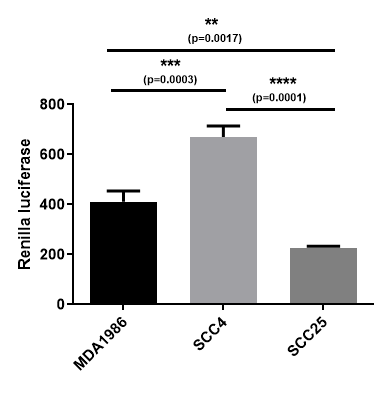
**

**C)**

**
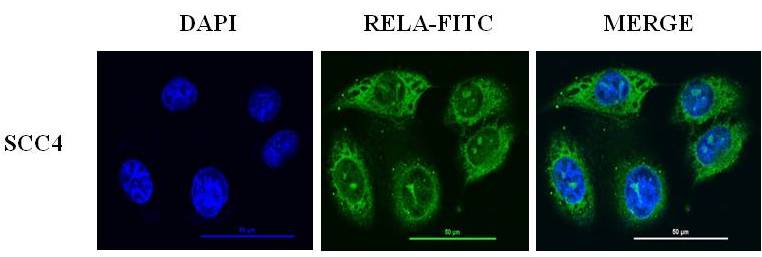
**

**Figure S1: (A) Renilla luciferase activity in various oral cancer cells co-transfected with pRL-TK to normalize for the variation in transfection efficiency. Values are mean ± SD from three independent experiments performed in triplicate. Results were analyzed using a ordinary one-way ANOVA.** **(B) Sub cellular fractionation was performed to isolate nuclear and cytosol fractions of SCC4 oral cancer cells. An equal amount of proteins from the fractions were resolved on 10% SDS-PAGE followed by western blotting using monoclonal antibodies against anti-RelA, anti-GAPDH and anti-Lamin B1. GAPDH and Lamin B1 were used as cytoplasmic and nuclear loading controls respectively. (C) Confocal Laser Scanning Microscopy was employed to detect the presence of RelA (green, FITC) in cytoplasmic and nuclear compartments. DAPI was used for staining the nuclear fraction.**


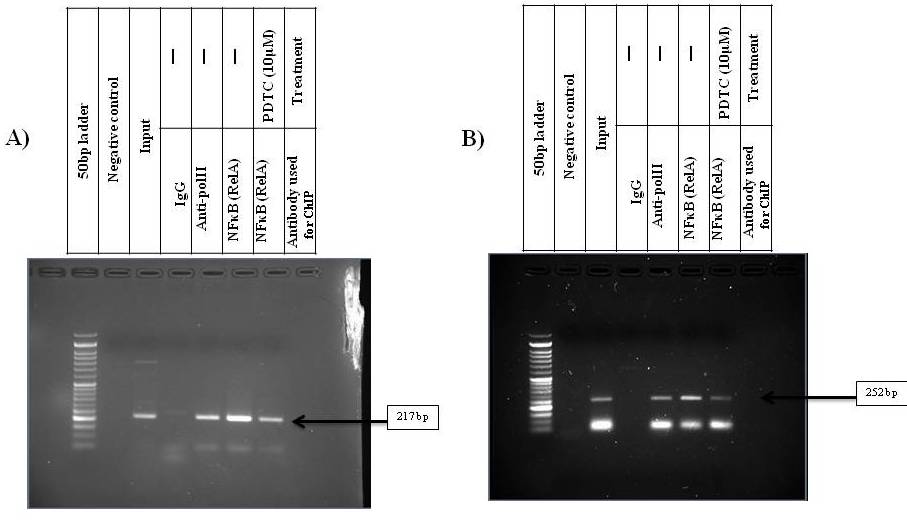


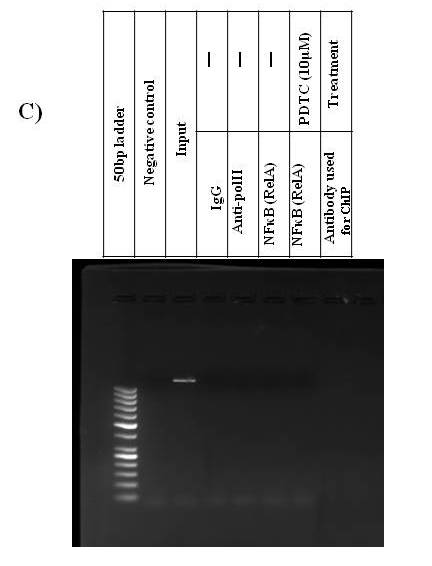


**Figure S2:** Full gel images of figure 6 A and 6 B represented as A and B respectively. (C) PCR was performed using hnRNPD ORF sense and antisense primers to hnRNPD gene as an additional negative control.

**Figure S3:** Full blot images of figure 5 A.
